# Comparison of the clinical effect of unilateral transverse process extrapedicular and bilateral transpedicular percutaneous kyphoplasty for thoracolumbar osteoporotic vertebral compression fracture

**DOI:** 10.3389/fsurg.2024.1395289

**Published:** 2024-07-18

**Authors:** Dingli Xu, Chaoyue Ruan, Yang Wang, Xudong Hu, Weihu Ma

**Affiliations:** ^1^ Health Science Center, Ningbo University Zhejiang, Ningbo, China; ^2^ Orthopedic Department, Ningbo No. 6 Hospital, Zhejiang, Ningbo, China

**Keywords:** thoracolumbar osteoporotic vertebral compression fracture, unilateral transverse process extrapedicular, percutaneous kyphoplasty, comparison study, surgery

## Abstract

**Background:**

Osteoporosis vertebral compression fractures (OVCF) are common with the aging process. This study aimed to compare the effects of unilateral transverse process extrapedicular (UEPKP) and bilateral transpedicular percutaneous kyphoplasty (BTPKP) for patients with thoracolumbar OVCF.

**Methods:**

Data from 136 patients with OVCF treated with single-level PKP in our hospital between May 2019 and April 2021 were studied. Patients were grouped based on surgical procedure: there were 62 patients in the UEPKP group and 74 in the BTPKP group. All clinical and radiological data were collected from medical records. Clinical outcomes, including visual analog scale (VAS), Oswestry Disability Index (ODI), and Japanese Orthopaedic Association (JOA) scores of the lumbar spine, were evaluated preoperatively, postoperatively, and at the follow-up visit. The radiological evaluations (anterior vertebral height rate and local kyphosis angle) and complications were also collected.

**Results:**

All patients had successfully improved after surgery. In the UEPKP group, patients showed a significantly shorter operating time and lower fluoroscopy frequency than patients in the BTPKP group (*p* < 0.05). However, a significantly better distribution score and cement volume were found in the BTPKP group (*p* < 0.05). The UEPKP group achieved a significantly better VAS score (0.6 ± 0.5 vs. 0.9 ± 0.8) and ODI (24.7 ± 3.1 vs. 27.5 ± 1.8) at the final follow-up visit than the BTPKP group (*p* < 0.05). The UEPKP group showed significantly worse radiological outcomes (anterior height rate and local kyphosis angle) at the 6- and 12-month follow-ups (*p* < 0.05). As for complications, the UEPKP group showed significantly fewer facet joint violations and intraspinal leakages (*p* < 0.05).

**Conclusion:**

UEPKP could be a safe and effective alternative procedure for patients with thoracolumbar osteoporotic vertebral compression fracture, which possesses an apparent advantage in reducing intraspinal leakage and facet joint violation over BTPKP.

## Introduction

Osteoporotic vertebral compression fractures (OVCF) have become common with the advent of the aging process, which places a huge burden on patients' health. Severe back pain, lumbar spine dysfunction, and even kyphosis may occur if OVCF is not treated in time (1). Wade et al. reported that the prevalence of osteoporosis in men and women is in the range of 3%–8% and 16%–38%, respectively, in industrialized countries (2). The anterior vertebral height of patients with OVCF may significantly decrease as a result of paravertebral muscle and ligament degeneration. Therefore, the main aims of treatment are as follows: (1) restore vertebral height and spinal realignment, (2) decrease the time to ambulation, and (3) relieve back pain (3). Recently, the bilateral transpedicular approach percutaneous kyphoplasty (BTPKP) has become the most common and widely used technique for OVCF as it relieves back pain immediately and restores vertebral alignment. Liu et al. conducted a meta-analysis study about bilateral transpedicular percutaneous kyphoplasty for OVCF and found that 16 studies involving 930 patients with OVCF achieved significantly rapid pain relief, spinal function improvement, vertebral height restoration, and kyphosis deformity correction after PKP (4).

However, some researchers found that patients treated with BTPKP may experience residual back pain and intraspinal leakage. Lin et al. found that 47 of 281 patients with OVCF had residual back pain after PKP. They proposed that facet joint violation and thoracolumbar fascia injury are the independent risk factors for residual low back pain (5). As for intraspinal leakage, in their case report, Li et al. reported that an 81-year-old female patient with OVCF underwent percutaneous kyphosis in L5, and a CT scan revealed a massive leakage of bone cement into the spinal canal (6). The transpedicular approach may increase the risks of facet joint violation, pedicle fracture, and intrude into the spinal canal, resulting in intraspinal leakage and residual back pain (7).

To avoid these risks of the transpedicular approach, Ryu et al. proposed unilateral extrapedicular percutaneous kyphoplasty (UEPKP) for patients with OVCF. In their study, 31 patients (37 vertebrae) were treated with UEPKP, and all patients achieved a significant improvement in visual analog scale (VAS), average middle body height, and local kyphotic deformity (*p* < 0.001) (8). Later, a less-demanding standardized UEPKP procedure was proposed by Ryu et al., and 29 patients with OVCF who underwent this new UEPKP procedure achieved significant improvement in VAS and cement distribution. Of the patients, 81.2% were satisfied (9).

However, few studies have compared the clinical effect between the unilateral extrapedicular approach and the bilateral transpedicular approach. Therefore, the aim of our study was to compare the clinical and radiological outcomes between UEPKP and BTPKP in patients with single-level thoracolumbar OVCF.

## Methods

### Patients

In total, 136 patients with single-level thoracolumbar OVCF who underwent UEPKP or BTPKP between May 2019 and April 2021 were enrolled in the study. The clinical and radiological outcomes of these patients were collected from medical records at Ningbo No. 6 Hospital. The inclusion criteria were as follows: (1) diagnosed as single-level thoracolumbar osteoporotic vertebral compression fracture by X-ray (T score < −2.5), (2) treated with UEPKP or BTPKP, (3) with a minimum follow-up of 1 year, and (4) complete data are available. The exclusion criteria were as follows: (1) history of spinal trauma and surgery, (2) spinal deformity or tumor, (3) multi-segment thoracolumbar fracture, or (4) diseases that incur bone mineral loss or bone destruction, including chronic renal failure and rheumatoid arthritis. Finally, 136 patients who met the inclusion and exclusion criteria were enrolled: 62 patients with unilateral extrapedicular percutaneous kyphoplasty were included in the UEPKP group and 74 patients who underwent bilateral transpedicular approach percutaneous kyphoplasty were added to the BTPKP group.

All procedures involving human participants were performed in accordance with the ethical standards of the institution, and the 1964 Declaration of Helsinki and its later amendments or comparable ethical standards. Informed consent was waived because of the retrospective nature of the study. The study was approved by the Institutional Review Board and Ethics Committee of Ningbo No. 6 Hospital.

### Operation procedure

The same senior spine surgeon performed all the operations on patients with OVCF. The patient was placed in the prone position, and their abdomen was suspended by bolsters under the chest and pelvis. The fractured vertebra was then located under the C-arm, and the projection of the surgical pedicle was marked on the skin surface. Local infiltration anesthesia was administered after disinfection and surgical draping.

### UEPKP group

**Figure 1:**
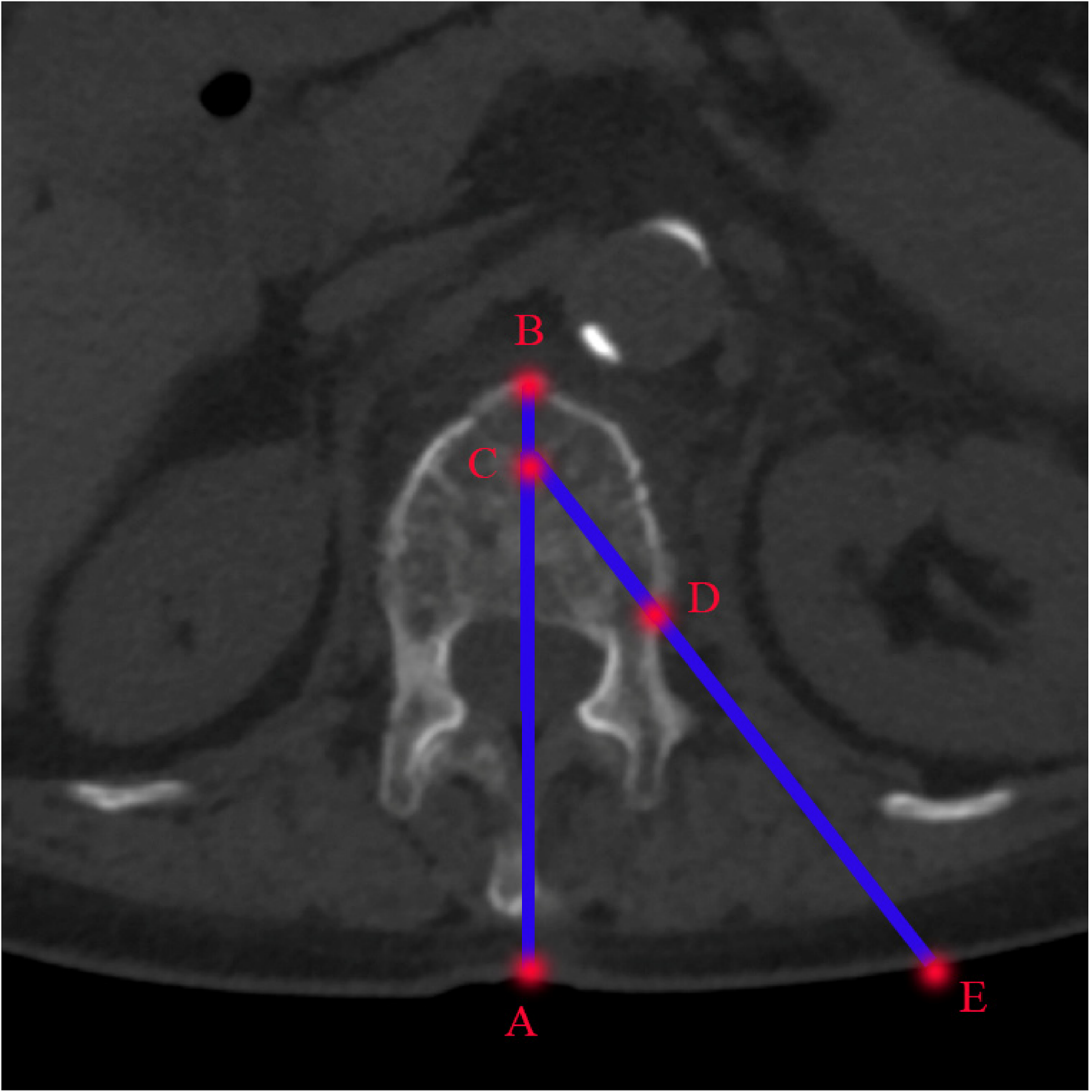
The measurement of puncture angle and distance based on preoperative cross-sectional CT scan. Line AB: middle line of the fractured vertebra; Point C: the anterior one-third point of the vertebral body on the middle line; Point D: the superolateral junction between the pedicle and the fractured vertebra. Point E: the intersection of line CD and skin surface. Line AE is the distance between the skin entry point and spinous process and Angle between lines AB and CE is the puncture angle.

**Figure 2:**
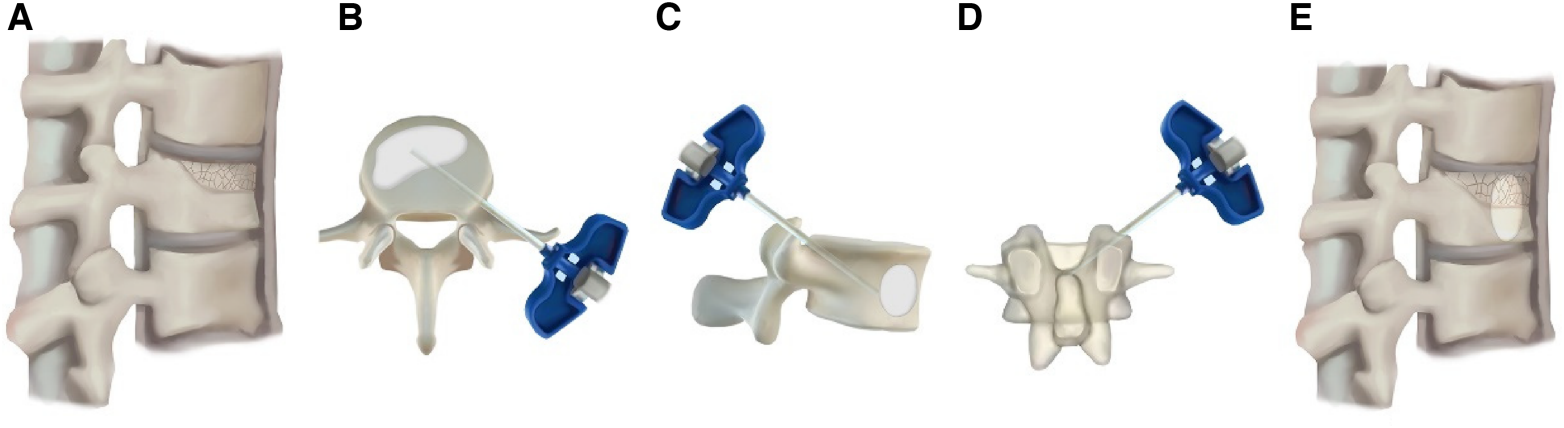
The operation procedures. (**A**) Vertebral compression fracture for which the anterior height was decreased; (**B**–**D**) UEPKP operation details; (**E**) the anterior height of the fractured vertebra was restored postoperatively.

Preoperatively, the puncture angle and distance between skin entry point and spinous process of fractured vertebra were measured based on preoperative CT scan (Figure 1). An incision of 0.5 cm was made based on the distance measured according to the preoperative CT scan and a puncture needle was inserted according to the puncture angle. The puncture needle was located at the superolateral junction between the pedicle and the fractured vertebra under C-arm fluoroscopy (Figure 2).

### BTPKP group

Two incisions of 0.5 cm were made approximately 2 cm lateral to the mark of each pedicle. Then a puncture needle was used to find the posterolateral aspect of the pedicle under C-arm guidance. Later, the puncture needle was advanced until it was located at the medial wall of pedicle on the anteroposterior radiograph and at the posterior wall of the vertebral body on the lateral radiograph.

The puncture needle was advanced approximately 2–3 cm and reached the anterior half to two-thirds of the vertebral body on the lateral radiograph. A working cannula and balloon retractor (<200 psi) were then inserted at the anterior quarter of the vertebrae. Polymethyl methacrylate bone cement was slowly injected via the working cannula under C-arm guidance until the anterior vertebral height was successfully restored. All patients were encouraged to ambulate early with a lumbar brace.

### Outcome evaluation

Baseline data, such as sex, age, surgery segment, body mass index (BMI), bone mineral density (BMD), fluoroscopy frequency, operation time, time to ambulation, cement volume, and distribution score, were collected from medical records.

Regarding the clinical symptoms of patients in pain and spinal function, VAS, Japanese Orthopaedic Association (JOA), and Oswestry Disability Index (ODI) were used in our study. Lumbar function was evaluated using the VAS for the back (0, no pain; 10, most severe pain), the ODI and JOA scores. McNab scores were used to evaluate patients’ satisfaction with the surgical result, which was divided into four categories: excellent, good, fair, and poor (10). Any complications, including residual back pain, facet joint violation, and cement leakage, were also recorded.

**Figure 3:**
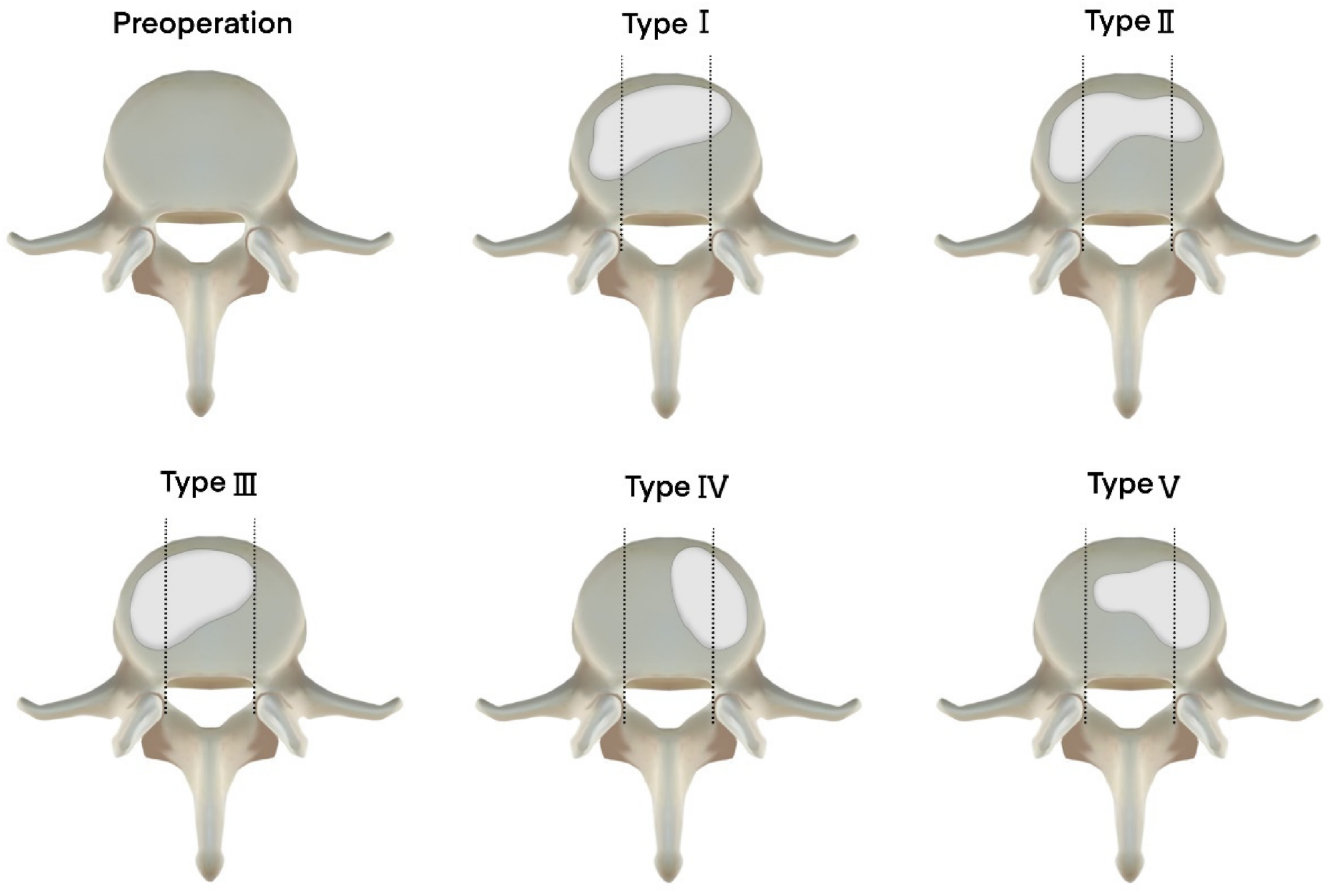
The classification of cement distribution type. Type I: evenly spread cement on both sides; type II: dominantly spread on the ipsilateral side and filled up sufficiently on the contralateral side; type III: dominantly spread on the ipsilateral side and filled up insufficiently on the contralateral side; type IV: only spread on the contralateral side; type V: dominantly spread on the contralateral side and filled up insufficiently on the ipsilateral side. This classification means that types I and II are satisfied cement distribution, and types III–V may cause vertebral collapse.

All the radiological outcomes were measured by two experienced radiologists. (1) Anterior height rate (AH): the ratio of the anterior to posterior height of the fractured vertebra based on a lateral spinal X-ray, calculated as anterior height/posterior height × 100%; (2) local kyphosis (LKA) angle of the vertebra: the Cobb angle between the superior and inferior endplates of the fractured vertebra; (3) the types of cement distribution were measured using the classification by Ryu et al. (Figure 3) (9); (4) the cement distribution score was evaluated using radiographs according to the method by Sun et al. (11). Precisely, the widths of the cement and operational vertebra were measured on anteroposterior X-rays by two experienced radiologists who then calculated the ratio of the widths. The length/height ratio was also measured. The score was then marked (3 points: ratio >75%; 2 points: ratio 50%–75%; 1 point: ratio 25%–50%; and 0 points: ratio <25%) and the total score was obtained by adding the three scores of width, length, and height.

### Statistical analysis

SPSS version 24.0 ( IBM Corp., Armonk, NY, USA) was used for statistical analysis. All data are expressed as the mean ± standard deviation. The Shapiro–Wilk test was used to determine the normality of continuous data. Age, BMI, ODI, and JOA data were compared using a *t*-test analysis. Categorical data were compared using χ^2^ tests. Statistical significance was set at *p* < 0.05.

## Results

### Baseline data

In total, 136 patients with single-level thoracolumbar osteoporotic vertebral compression fractures who underwent UEPKP or BTPKP at our hospital between May 2019 and April 2021 were enrolled in the study. As shown in Table 1, there were no significant differences in age, gender, BMD, BMI and operation segment between the 62 patients in the UEPKP group and the 74 patients in the BTPKP group (*p* > 0.05). In addition, a significantly shorter operation time (35.9 ± 3.3 min vs. 44.9 ± 3.8 min, *p* < 0.05) and lower fluoroscopy frequency (30.0 ± 4.3 vs. 38.5 ± 2.6, *p* < 0.05) were observed in the UEPKP group compared with the BTPKP group. However, patients in the BTPKP group achieved significantly better distribution scores (7.9 ± 0.7 vs. 6.9 ± 0.8, *p* < 0.05) and cement volumes (5.0 ± 0.3 vs. 4.2 ± 0.5 ml, *p* < 0.05) than patients in the UEPKP group, whereas there were no significant difference in distribution type between the two groups (*p* > 0.05).

**Table 1:** Comparison of baseline and operation-related outcomes between the two groups.

Characteristic	UEPKP group	BTPKP group	*p*-value
Cases	62	74	
Age (years)	69.4 ± 5.2	68.8 ± 7.8	0.66
Gender			
Male	25	32	0.73
Female	37	42	
BMD	−3.0 ± 0.3	−2.9 ± 0.5	0.19
BMI (kg/m^2^)	22.6 ± 1.8	22.9 ± 2.1	0.57
Fluoroscopy frequency	30.0 ± 4.3	38.5 ± 2.6	<0.05*
Operation time (min)	35.9 ± 3.3	44.9 ± 3.8	<0.05*
Operation segment			
T11	3	9	0.32
T12	23	26	
L1	28	30	
L2	8	9	
Time to ambulation (days)	1.4 ± 0.5	1.5 ± 104	0.85
Distribution score	6.9 ± 0.8	7.9 ± 0.7	<0.05*
Cement volume (ml)	4.2 ± 0.5	5.0 ± 0.3	<0.05*
Distribution type			0.16
I	37	58	
II	18	12	
III	4	2	
IV	1	0	
V	2	2	

* *P* < 0.05.

### Clinical outcome

Table 2 presents the clinical outcomes of the two groups. There were no significant differences between the two groups in VAS, JOA, and ODI preoperatively (*p* > 0.05). During the follow-up visit, there were no significant differences in VAS between the UEPKP and BTPKP groups but the UEPKP group achieved significantly better pain relief at the 12-month follow-up (0.6 ± 0.5 vs. 0.9 ± 0.8, *p* < 0.05). As for JOA score, there was no significant difference between the two groups at every follow-up (*p* > 0.05). However, a significant improvement in ODI score (24.7 ± 3.1 vs. 27.5 ± 1.8, *p* < 0.05) was observed at the final follow-up in the UEPKP group compared with the BTPKP group. In the UEPKP group, according to the McNab score, 40 patients were excellent, 19 were good, 2 were fair, and 1 was poor at the final follow-up. In the BTPKP group, 35 patients were excellent, 24 were good, 10 were fair, and 5 were poor. The difference between the two groups was statistically significant (*p* < 0.05).

**Table 2:** Comparison of clinical outcomes between the two groups.

Characteristic	UEPKP group	BTPKP group	*p*-value
VAS			
Preoperation	7.0 ± 0.7	6.8 ± 0.8	0.35
1 day	2.9 ± 0.9	2.8 ± 0.8	0.51
1 month	2.4 ± 0.5	2.6 ± 0.6	0.19
6 months	1.5 ± 0.5	1.6 ± 0.6	0.49
12 months	0.6 ± 0.5	0.9 ± 0.8	<0.05*
JOA			
Preoperation	12.3 ± 1.8	12.5 ± 1.5	0.44
1 month	19.4 ± 2.1	19.1 ± 2.5	0.57
6 months	22.9 ± 1.3	22.8 ± 1.6	0.71
12 months	23.6 ± 1.0	23.9 ± 1.5	0.29
ODI			
Preoperation	70.7 ± 4.3	71.5 ± 5.4	0.39
1 month	36.6 ± 3.6	38.1 ± 5.7	0.07
6 months	28.9 ± 2.6	28.2 ± 3.5	0.23
12 months	24.7 ± 3.1	27.5 ± 1.8	<0.05*
McNab			<0.05*
Excellent	40	35	
Good	19	24	
Fair	2	10	
Poor	1	5	

* *P* < 0.05.

### Radiological outcomes

As shown in Table 3, the preoperative AH (69.2% ± 4.5% vs. 68.8% ± 2.6%) and LKA (14.4° ± 1.3° vs. 14.6° ± 1.3°) were similar between the UEPKP and BTPKP groups (*p* > 0.05). Both groups achieved significant recovery in AH and LKA postoperatively; however, the BTPKP group showed significantly better AH (89.5% ± 3.1% vs. 90.4% ± 1.9% and 86.4% ± 2.1% vs. 88.9% ± 1.5%) and LKA (9.8° ± 0.7° vs. 9.3° ± 0.6° and 9.7° ± 0.6° vs. 9.2° ± 0.8°) at the 6- and 12-month follow-ups, respectively (*p* < 0.05).

**Table 3:** Comparison of radiological outcome and complication between the two groups.

Characteristic	UEPKP group	BTPKP group	*p*-value
AH			
Preoperation	69.2 ± 4.5	68.8 ± 2.6	0.45
1 day	94.6 ± 0.9	94.7 ± 1.7	0.58
1 month	91.7 ± 1.5	91.9 ± 1.2	0.17
6 months	89.5 ± 3.1	90.4 ± 1.9	<0.05*
12 months	86.4 ± 2.1	88.9 ± 1.5	<0.05*
LKA			
Preoperation	14.4 ± 1.3	14.6 ± 1.3	0.39
1 day	6.9 ± 0.9	6.6 ± 1.0	0.18
1 month	8.3 ± 0.4	8.2 ± 0.6	0.43
6 months	9.8 ± 0.7	9.3 ± 0.6	<0.05*
12 months	9.7 ± 0.6	9.2 ± 0.8	<0.05*
Complication			
Residual back pain	0	4	<0.05*
Facet joint violation	0	8	<0.05*
Intraspinal leakage	0	5	<0.05*
Paravertebral leakage	5	6	0.62
Intervertebral leakage	1	2	0.56

* *P* < 0.05.

### Complications

As for complication, six patients experienced cement leakage in the UEPKP group and were diagnosed using CT (five with paravertebral leakage and one with intervertebral leakage). No further treatment was given to these six patients as they had no clinical symptoms. In the BTPKP group, four patients had residual back pain that was successfully managed with oral non-steroidal anti-inflammatory drugs and traditional Chinese manipulation and acupuncture. Eight patients were diagnosed with facet joint violation and 13 patients had cement leakage diagnosed via radiography. There was a statistically significant difference between the two groups in complications (*p* < 0.05).

## Discussion

Transpedicular percutaneous kyphoplasty was first proposed by Garfin et al. They reported that 340 patients with OVCF who were treated with this method achieved significant improvement in anterior vertebral height (from 68° ± 12° to 84° ± 14°, *p* < 0.01) and middle vertebral height (from 64° ± 13° to 90° ± 12°, *p* < 0.01) (12). For the last two decades, transpedicular percutaneous kyphoplasty has been widely used for the treatment of OVCF because of its satisfying clinical effect. There are many published studies that have analyzed and compared the effect of transpedicular percutaneous kyphoplasty. Masoudi et al. conducted a randomized clinical trial about the comparison between percutaneous kyphoplasty and conservative treatment for 70 patients with thoracolumbar fractures (type A1 and A2) (13). They found that patients treated with percutaneous kyphoplasty achieved significantly better improvement in VAS and ODI scores at follow-up visits than patients who underwent conservative treatment (*p* < 0.001). Moreover, a significantly shorter period of absence from work (49.8 ± 12.5 vs. 73.5 ± 12.4 days, *p* < 0.05) was also observed in patients who had percutaneous kyphoplasty (13). In the same vein, Yu et al. conducted a comparative study between transpedicular PKP and conservative treatment in elderly patients with OVCF (14). They found that the PKP group achieved significantly better results for pain relief, vertebral height restoration, and enhanced spinal function (*p* < 0.05) (14).

However, the drawbacks of transpedicular PKP have also been reported, including residual back pain, facet joint violation, and cement leakage. Yang et al. reported that 19 of 98 patients with single-level OVCF who underwent PKP experienced residual back pain (VAS score >4 at 6 months postoperatively) (15). Similarly, Li et al. conducted a study to analyze the risk factors of residual back pain in OVCF patients after PKP. Based on a multivariate logistic regression analysis of the data of 52 eligible patients, the possible risk factors of residual back pain were facet joint violations [odds ratio (OR) 12.19, *p* < 0.001], intravertebral vacuum cleft (OR 2.93, *p* = 0.032), and posterior fascia edema (OR 4.11, *p* = 0.014) (16). As for cement leakage, Sun et al. reported that 52 patients with OVCF underwent PKP and 11 patients were diagnosed with cement leakage (5 had intraspinal cement leakage) (17). With regard to cement leakage perioperatively, Zhang et al. reported a conception of safety line that was supposed to prevent intraspinal cement leakage during percutaneous kyphoplasty. However, 7 of 80 patients who were treated with PKP using the safety line still experienced intraspinal cement leakage (18).

The transpedicular approach could be the main cause of facet joint violation and residual back pain (19). UEPKP for OVCF was proposed to avoid facet joint violation and residual back pain. Zhu et al. evaluated the clinical effect of UEPKP for patients with lumbar OVCF and reported that 48 patients who were treated with UEPKP achieved a significant decrease in postoperative VAS (7.9 ± 0.9 vs. 2.9 ± 0.7, *p* < 0.05) and ODI (73.3 ± 8.1 vs. 33.7 ± 5.4, *p* < 0.05) scores (20). Similarly, Ge et al. reported that 38 thoracic OVCF patients were treated with unilateral extrapedicular approach PKP, and significantly improvement in VAS (8.92 ± 0.68 vs. 2.40 ± 0.31, *p* < 0.05) and correction in vertebral anterior height (18.55 ± 4.32 vs. 22.90 ± 4.57, *p* < 0.05) were observed (21).

Our study compared the clinical and radiological outcomes between the UEPKP and BTPKP groups. At the 1-year follow-up, the UEPKP group had a significant improvement in pain relief and spinal function (VAS and ODI) (*p* < 0.05). Furthermore, the UEPKP group also achieved a significantly better McNab score than the BTPKP group (*p* < 0.05). Compared with BTPKP group, the fluoroscopy frequencies (30.0 ± 4.3 vs. 38.5 ± 2.6, *p* < 0.05) and operation time (35.9 ± 3.3 min vs. 44.9 ± 3.8 min, *p* < 0.05) were both better in the UEPKP group. However, the BTPKP group showed a significantly improved cement volume (5.0 ± 0.3 vs. 4.2 ± 0.5 ml, *p* < 0.05) and distribution score (7.9 ± 0.7 vs. 6.9 ± 0.8, *p* < 0.05) than the UEPKP group. In addition, the BTPKP group showed a significantly better anterior height rate and local kyphosis angle at the 6- and 12-month follow-ups, but the clinical significance of the difference in radiological outcomes is not obvious. Moreover, significantly more complications were observed in the BTPKP group (four patients experienced residual back pain, eight patients had facet joint violation, and five had intraspinal cement leakage) than the UEPKP group (*p* < 0.05). Similar outcomes were also reported by Zhu et al. They conducted a comparison between unilateral extrapedicular (34 patients) and bilateral transpedicular (42 patients) percutaneous kyphoplasty in patients with lumbar OVCF and found that patients in the unilateral extrapedicular group showed significantly better fluoroscopy times (21.6 ± 3.5 vs. 34.2 ± 2.4, *p* < 0.01), operation times (35.6 ± 4.6 vs. 46.2 ± 8.8 min, *p* < 0.01), and facet joint violation (11 vs. 0, *p* < 0.01) than patients in the bilateral transpedicular group. Both groups achieved significant improvements in VAS, ODI, vertebral height, and local kyphosis angle postoperatively, but there were no significant differences between the two group during the follow-up visit (*p* > 0.05) (22). Yan et al. also carried out a prospective study about the comparison between these two surgical methods (158 patients in the unilateral group and 151 in the bilateral group). They discovered that 8 patients experienced intraspinal cement leakage and 16 patients experienced residual back pain that was caused by facet joint violation in the bilateral group, whereas only 1 patient had intraspinal cement leakage in the unilateral group (23). UEPKP can reduce the incidence of facet joint violation during surgery because the entry point and trajectory are different from the traditional bilateral transpedicular approach. In addition, UEPKP can lessen approach-related complications, including pedicle fractures and posterior structure of vertebrae damage, which can result in intraspinal cement leakage (24).

In conclusion, the UEPKP group achieved significant improved pain relief and spinal function compared with the BTPKP group, and UEPKP reduced the operating time, fluoroscopy frequencies, and rate of complications for patients with thoracolumbar OVCF. UEPKP can reduce operation time and fluoroscopy frequencies compared with BTPKP, because only one approach is needed during surgery, which is convenient for puncture and C-arm fluoroscopy. In addition, because of the approach advantage, UEPKP can effectively prevent damage to the posterior structure of the vertebra, pedicle, and facet joint, which could be a possible reason for less residual back pain and intraspinal cement leakage. As for selecting the left or right side during UEPKP, the following points need to be considered: (1) the pedicle of OVCF, which is complete without fracture; (2) the width of the pedicle; and (3) the anterior height rate of the fractured vertebra.

However, the BTPKP group showed a significantly better anterior height rate and local kyphosis angle compared with the UEPKP group. A reason for this could be that there was significantly more cement volume and higher distribution score in the BTPKP group. Therefore, the patients in the BTPKP group can achieve better reduction and anterior vertebral support from cement. This is why the BTPKP group achieved better radiological outcomes than the UEPKP group.

## Limitations

This study has some limitations. First, our study was vulnerable to bias because of its retrospective nature, but we minimized bias by enrolling patients using inclusion and exclusion criteria. Second, we only analyzed the clinical and radiological outcomes within the 1-year follow-up visit, and vertebral collapse or refracture may occur if the follow-up is extended. Finally, because of the small sample size, we did not take subgroups into account, such as patients with severe osteoporosis or very elderly patients.

## Data Availability

The original contributions presented in the study are included in the article/Supplementary Material, further inquiries can be directed to the corresponding author.
